# The Role and Function of TRPM8 in the Digestive System

**DOI:** 10.3390/biom14070877

**Published:** 2024-07-21

**Authors:** Zunan Wu, Shuai Peng, Wensha Huang, Yuling Zhang, Yashi Liu, Xiaoyun Yu, Lei Shen

**Affiliations:** 1 Department of Gastroenterology, Renmin Hospital of Wuhan University, Wuhan 430060, China; 2018305231083@whu.edu.cn (Z.W.); pengpengspace@whu.edu.cn (S.P.); 2019305232103@whu.edu.cn (W.H.); 2 Hubei Key Laboratory of Digestive Diseases, Wuhan 430060, China; 3 Department of Gastroenterology, Union Hospital, Tongji Medical College, Huazhong University of Science and Technology, Wuhan 430022, China; m202275936@hust.edu.cn (Y.Z.); m202376102@hust.edu.cn (Y.L.)

**Keywords:** TRPM8, digestive system, menthol

## Abstract

Transient receptor potential (TRP) melastatin member 8 (TRPM8) is a non-selective cation channel that can be activated by low temperatures (8–26 °C), cooling agents (including menthol analogs such as menthol, icilin, and WS-12), voltage, and extracellular osmotic pressure changes. TRPM8 expression has been identified in the digestive system by several research teams, demonstrating its significant involvement in tissue function and pathologies of the digestive system. Specifically, studies have implicated TRPM8 in various physiological and pathological processes of the esophagus, stomach, colorectal region, liver, and pancreas. This paper aims to comprehensively outline the distinct role of TRPM8 in different organs of the digestive system, offering insights for future mechanistic investigations of TRPM8. Additionally, it presents potential therapeutic targets for treating conditions such as digestive tract inflammation, tumors, sensory and functional disorders, and other related diseases. Furthermore, this paper addresses the limitations of existing studies and highlights the research prospects associated with TRPM8.

## 1. The Structure, Functions, and Distribution of TRPM8

The TRP channel is a type of non-selective cation channel located on the cell membrane, comprising various subtypes including TRPC (canonical), TRPV (vanilloid), TRPM (melastatin), TRPA (ankyrin), TRPP (polycystin), TRPML (mucolipin), and TRPN (*Drosophila* NOMPC) [1]. TRPM8 stands out as one of the extensively studied TRPM channels and allows the permeation of monovalent and divalent cations, such as Na^+^, K^+^, and Ca^2+^, with a higher permeability to Ca^2+^ [2]. Structurally, this channel consists of a tetrameric protein with six transmembrane domains (S1-S6) and intracellular N-terminal and C-terminal segments [3]. The S2-S4 regions harbor a menthol binding site of high affinity [4], while the S3 domain possesses amino acids sensitive to icilin [5]. Additionally, the voltage sensor is located at the connection of the S4 segment and the S4-S5 domains [6], forming a channel pore across transmembrane segments S5 and S6 [7]. Consequently, the channel can be activated by various stimuli, including low temperatures (8–26 °C); coolants like menthol, icilin, and WS-12 (menthol analogs); voltage changes; and modifications in extracellular osmotic pressure [8,9].

Initially isolated from prostate cancer cells, TRPM8 channels were primarily investigated in relation to prostate cancer, highlighting their protective anti-invasion properties and their potential as diagnostic and prognostic markers. However, expanding research has unveiled the wide distribution of TRPM8 throughout the human body [10,11,12]. In addition to the prostate, TRPM8 is identified in organs such as the pancreas, testes, breasts, thymus, lungs, seminiferous tubules, skin, bladder, liver, brain, colon, sperm cells, nasal mucosa, and cilia [13].

Prior studies have extensively documented the functional and structural aspects of TRPM8 and its involvement in critical physiological processes, including inflammatory responses, carcinogenesis, cell proliferation, immune modulation, and thermoregulation. Moreover, research on TRPM8 has ventured into new domains such as the nervous system, digestive system, respiratory system, and skin [3,14,15,16]. Particularly noteworthy is the significant role of TRPM8 in either promoting or inhibiting various digestive tract-related diseases (Table 1). Investigations focusing on TRPM8 in esophageal, gastric, intestinal, hepatic, and pancreatic disorders mainly revolve around inflammation, cancer, sensory responses, and motility. This paper aims to consolidate the advancements in TRPM8 research within the digestive system, aiming to enhance the comprehension of the functionality of the TRPM8 channel; provide insights for further exploration of the underlying mechanisms in digestive diseases; and present potential targets and markers for the clinical diagnosis, treatment, and prognosis of digestive disorders.

## 2. The Role of TRPM8 in the Esophagus

TRPM8 plays a crucial role in various physiological and pathological processes such as esophageal sensation, motility, inflammation, and cancer. Studies have linked esophageal sensory functions to the vagus nerve, specifically involving high-threshold nociceptive vagus nerve afferent subtypes—namely, vagus nerve nodal C fibers and jugular C fibers—with TRPM8 expression identified in sensory neurons of the vagus nerve [46,47]. Researchers observed that menthol and cold stimulation can induce esophageal sensory dysfunction, potentially through TRPM8 activation, substantiating the presence of TRPM8 in jugular neurons while being absent in the nodular neurons. The isolation of guinea pig esophageal vagus nerve jugular and nodular neurons enabled a comprehensive study. The patch-clamp technique revealed that the TRPM8 agonist WS-12 selectively evoked action potential discharges in the jugular but not nodose C fiber nerve endings in the esophagus, affirming the role of TRPM8 activation in esophageal sensory function mediation via jugular neurons in the esophageal vagus nerve [17].

Moreover, TRPM8 is implicated in regulating esophageal motility. An early study showed that the oral intake of peppermint oil can reduce spasm of the esophagus [48]. Recent advancements in esophageal motility assessment, particularly through high-resolution manometry, revealed that cold water prolonged the esophageal contraction duration and reduced the peristalsis amplitude in healthy individuals [49]. Another study highlighted that menthol activation significantly lowered the upper esophageal pressure during primary peristalsis and inhibited the secondary peristalsis frequency from rapid air expansion, suggesting a role for TRPM8 in esophageal motility regulation [50]. Notwithstanding the absence of direct evidence linking TRPM8 to esophageal motility, the capacity of hypothermia and menthol to activate TRPM8 implies its involvement in this process. Further inquiry into the molecular underpinnings of esophageal spasm may yield insights for treating spasm-related pain and gastroesophageal reflux disease through TRPM8 modulation.

Notably, TRPM8 exhibits a promising anti-inflammatory effect in the esophagus. Prior investigations underscored the pivotal role of TRPV1 in esophageal sensation and inflammation, where acid-induced stimulation intensified esophageal pain and inflammation via enhanced TRPV1 expression and cell proliferation in esophageal epithelial cells [51,52,53]. In light of menthol’s inhibitory influence on TRPV1 and gastric acid secretion [54,55], this study examined whether menthol could ameliorate esophageal inflammation through TRPV1 inhibition. They discovered that menthol impeded the proliferation of esophageal epithelial cells, reduced TRPV1 expression in esophageal epithelial cells both in vivo and in vitro, and ameliorated pain and inflammation associated with reflux esophagitis [56]. Building on the findings regarding the inhibitory impact of TRPM8 on TRPV1 in analogous research [24,33], it is anticipated that TRPM8 may emerge as a promising target for reflux esophagitis treatment.

In esophageal cancer, TRPM8 has been implicated in promoting tumor cell proliferation and immune evasion. Research has demonstrated a higher TRPM8 mRNA expression in human esophageal cancer tissues compared to adjacent tissues and an elevation in human esophageal cancer cell lines compared to normal esophageal epithelial cells, with the knockdown or inhibition of TRPM8 resulting in a reduced cancer cell proliferation, while the overexpression of TRPM8 had the opposite effect. Mechanistically, this effect might be linked to programmed death ligand 1 (PD-L1) expression induced by the calcineurin–nuclear factor of activated T cells 3 (NFATc3) pathway [18]. Globally, esophageal cancer ranks fourth among the most prevalent and lethal digestive system malignancies. Despite surgical interventions being the primary treatment option, effective pharmacological therapies are urgently warranted in clinical practice. Elucidating the regulatory mechanisms of TRPM8 in esophageal cancer holds promise for advancing the diagnosis and management of this disease [57,58,59].

## 3. The Role of TRPM8 in the Stomach

TRPM8 exerts a distinctive influence in the stomach, as its expression can be detected within the stomach mucosa and subsets of vagal afferent neurons that project to the stomach [47]. Through clinical assessments, researchers observed significantly elevated levels of TRPM8 protein expression in gastric cancer tissues compared to adjacent healthy tissues. This enhanced expression correlated significantly with tumor diameter, lymph node metastasis, and distal cancer cell metastasis. Patients with heightened TRPM8 protein expression exhibited a markedly lower 5-year overall survival rate compared to those exhibiting low TRPM8 protein expression. The presence of TRPM8 in patients with metastasis markedly exceeded that in individuals without metastasis, suggesting its potential role in promoting gastric cancer cell proliferation and metastasis [19]. Mechanistically, TRPM8 activation triggers Ca^2+^ influx into cells, initiating cascades of biological responses including cell proliferation, differentiation, and migration [60,61]. Further elucidation of the precise molecular pathways is essential for future investigations.

Despite the availability of tumor markers like carcinoembryonic antigen (CEA), CA19-9, CA72-4, CA125, and CA242 for gastric cancer diagnosis, their effectiveness in detecting early-stage disease remains limited, with early gastric cancer detection rates as low as 10%. There is an urgent demand for developing routine, non-invasive, highly specific biomarkers for early detection and treatment guidance in gastric cancer [62,63,64]. A study reported the RNA sequencing of tumor samples from 21 gastric cancer patients and identified 30 representative genes from 3192 differentially expressed genes, with subsequent validation through Western blotting and immunohistochemical techniques revealing significantly elevated TRPM8 expression levels in tumor tissues compared to normal gastric tissue. This identifies TRPM8 as a potential novel biomarker for gastric cancer diagnosis and treatment guidance, while also unveiling its potential as a therapeutic target [20].

Moreover, TRPM8 may participate in physiological mechanisms associated with anti-apoptosis, anti-oxidation, and anti-inflammation in the stomach. Research shows that menthol intervention in a rat model of an ethanol-induced gastric ulcer demonstrated a protective impact on stomach health, evidenced by alterations in myeloperoxidase (MPO), glutathione (GSH), glutathione peroxidase (GSH-Px), glutathione reductase (GR), superoxide dismutase (SOD), IL-6, IL-10, and TNF-α levels [65]. However, the involvement of TRPM8 in these responses is not explicitly verified in this study, warranting further dedicated investigations on whether menthol exerts these effects through TRPM8.

## 4. The Role of TRPM8 in the Small Intestine and Colon

TRPM8 exerts multifaceted effects on the gut, influencing sensory, motor, inflammatory, and tumor-related processes, with a growing body of mechanistic studies providing deeper insights. For instance, in terms of intestinal inflammation, TRPM8 exhibits an anti-inflammatory role, but the underlying mechanisms vary. According to research, the mitigating effect of WS-12 on small intestine inflammation was confirmed, followed by investigations in wild-type and TRPM8 gene knockout mice post-indomethacin stimulation. Remarkably, TRPM8 gene knockout mice exhibited increased intestinal damage compared to wild-type counterparts. Additionally, the upregulation of CGRP was noted in wild-type mice but not in TRPM8 knockout mice, with both groups displaying elevated SP expression. Consequently, it is proposed that TRPM8 may shield against indomethacin-induced small intestine injury via CGRP upregulation, independent of SP mediation [21].

SP and CGRP are two neuropeptides with distinct functions. SP is recognized for its role in immune system regulation and the exacerbation of intestinal mucosal inflammation, often co-expressed with TRPM8 in the distal colonic nerve [34,66]. On the other hand, CGRP is acknowledged to modulate immune cell activity and is typically perceived as a pro-inflammatory neuropeptide capable of fostering inflammatory responses. Nevertheless, observations of CGRP’s protective effects in colitis were documented, suggesting that its efficacy may be contingent on factors like release concentration, receptor expression, affinity, and the temporal pattern of neurosecretory activity associated with the inflammatory cascade [67,68].

**Figure 1:**
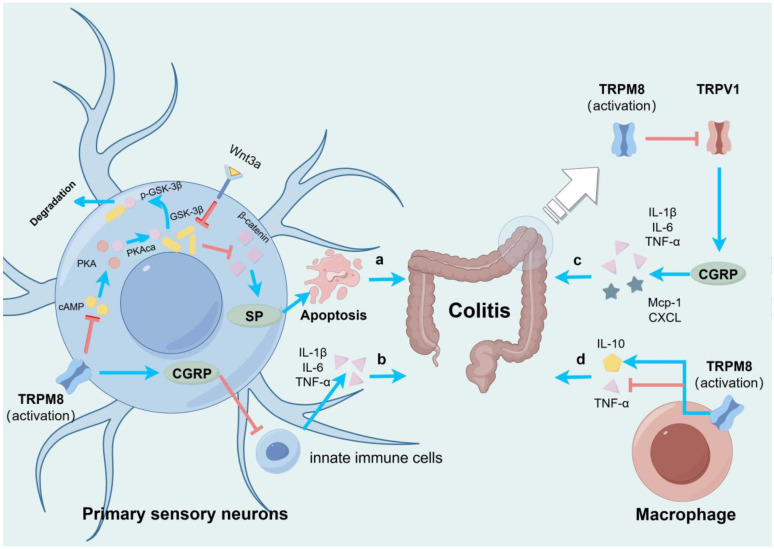
TRPM8 plays an anti-inflammatory role in colitis. Its mechanisms are as follows: (a) TRPM8 disrupts the PKAca and GSK-3β interaction, thereby impeding the SP apoptotic impact on colonic epithelial cells and mitigating colonic inflammation. (b) TRPM8 upregulates CGRP to suppress the production of pro-inflammatory cytokines by innate immune cells. (c) TRPM8 inhibits TRPV1 and attenuates the pro-inflammatory action of CGRP. (d) TRPM8 modulates the expression of IL-10 and TNF-α to reduce colonic inflammation. The blue lines represent “promote”, and the red lines represent “inhibit” in Figure 1.

The dual effects of CGRP are prominently examined in various colitis studies. A study shows that TRPM8 curbs the release of SP from primary sensory neurons by disrupting the protein kinase A catalytic subunit α (PKAca) and glycogen synthase kinase-3 beta (GSK-3β) interaction, hampering SP’s apoptotic influence on colonic epithelial cells and dampening colonic inflammation. The detection and validation of TRPM8 expression were confirmed using the TRPM8 antagonist N-(3-aminopropyl)-2-[(3-methylphenyl) methoxy]-N-(2-thienylmethyl) benzamide hydrochloride (AMTB hydrochloride). To be exact, menthol-induced calcium influx in mouse dorsal root ganglion cells suppressed PKAca binding and phosphorylation in the cyclic AMP (cAMP) signaling pathway to GSK-3β. GSK-3β serves as the primary inhibitory factor in the Wnt/β-catenin signaling pathway. It undergoes degradation following phosphorylation. In contrast, unphosphorylated GSK-3β can attenuate the promotion of SP release from primary sensory neurons by Wnt3a-driven β-catenin [22]. In contrast, another experiment proposed that TRPM8 counteracts inflammation by upregulating CGRP instead of inhibiting SP. This modulation may involve CGRP suppressing pro-inflammatory cytokine (IL-1β, IL-6, and TNF-α) production by innate immune cells such as CD11c+ dendritic cells. This study initially highlighted TRPM8’s protective role in acute colitis, emphasizing that TRPM8-deficient or CGRP-receptor-deficient mice exhibited exacerbated inflammation, a discrepancy not evident in SP receptor deficiency. Notably, CGRP administration mitigated colonic inflammation, demonstrating TRPM8/CGRP co-expression in human and wild-type mouse colonic mucosal fibers, accentuated during colitis. Nevertheless, evidence supporting TRPM8 activation eliciting CGRP upregulation is scarce. Rather, the accumulation of CGRP was observed in the colons of TRPM8^-/-^ mice, purportedly attributed to heightened inflammation post-dextran sodium sulfate (DSS) induction [23]. In addition, other experiments also identified augmented CGRP levels in TRPM8-deficient colitis mice. They suggest that CGRP may induce a pro-inflammatory effect, while TRPM8 could potentially mitigate the release of inflammatory cytokines (IL-1β, IL-6, and TNF-α) and inflammatory chemokines (monocyte chemoattractant protein-1 (MCP-1) and CXC chemokine ligand (CXCL)) by inhibiting the release of CGRP through TRPV1 [24]. Another study indicates that TRPM8’s anti-inflammatory function may not hinge on neuropeptides but is primarily mediated through systemic IL-10 promotion and regulating tumor necrosis TNF-α suppression. Notably, there were no alterations in neuropeptide levels, including CGRP or SP, in the experimental findings [25]. Although consistent anti-inflammatory effects of TRPM8 in colitis are observed across studies, further research is warranted to elucidate the precise underlying mechanisms (Figure 1).

Furthermore, TRPM8 is implicated in colon cancer, playing a pivotal role in the initiation, progression, and metastasis of the disease. The expression of TRPM8 in colon cancer has been elucidated, showcasing the potential inhibition of colon cancer onset by the TRPM8 antagonist cannabinol. This inhibition can impede the advancement of colon cancer, selectively restraining the proliferation of colorectal cancer cells, an effect shared by other TRPM8 antagonists [10,26]. Recent pharmacological investigations have validated that TRPM8 blockade can diminish tumor growth in colorectal cancer xenograft mice by downregulating the transcription of Wnt signaling regulators and tempering the activation of β-catenin, alongside its target oncogenes like *C-Myc* and *Cyclin D1*. Additionally, an investigation into the relationship between TRPM8 expression and the survival rates of colorectal cancer patients revealed a lower survival rate among those with elevated TRPM8 expression [27].

In addition to its association with colon cancer, TRPM8 also plays a role in the prevalent liver metastasis of colon cancer. A heightened expression of TRPM8 in human colon cancer has been shown, particularly in cases with liver metastasis. The in vitro investigations of one study validated the increased TRPM8 expression in colon cancer cells and further explored a model for colon cancer liver metastasis. The authors recorded a significant decrease in clone number, cell invasion, migration capabilities, and liver metastasis proportion following TRPM8 gene silencing, indicating that TRPM8 upregulation may foster colon cancer liver metastasis. This phenomenon might be linked to TRPM8 gene silencing, inhibiting the protein kinase B (Akt)/GSK-3β pathway, thereby impeding colon cancer cell progression, EMT, and subsequent liver metastasis [28].

Conversely, certain studies propose that TRPM8 inhibits the liver metastasis of colorectal cancer. Research indicates that TRP channel-associated factor 2 (TCAF2) in tumor pericytes (TPC) inhibits TRPM8 and promotes Wnt5a secretion through TRPM8 activation or inhibition, overexpression, or knockout experiments, and then activates the signal transducer and activator of transcription 3 (STAT3) signal pathway in tumor cells to promote EMT and realize liver metastasis of colorectal cancer [29]. Integral to these processes, TPC functions as a strategic vascular component within the blood vessel lumina, serving as the guardian of tumor enforcements [69]. Furthermore, TCAF2 acts as a crucial TRPM8 chaperone within the TRP cation channel subfamily. Prior research on prostate cancer has unearthed parallel findings, where TCAF2’s interaction with TRPM8 facilitates its cell surface transport and suppresses its ion channel activity, effectively enhancing the migration of prostate cancer cells in vitro [14,70]. Two experiments observed that the opposite function of TRPM8 may differ from its function in different tissues. Consequently, TRPM8 emerges as a prospective prognostic, tumor, and therapeutic marker for both colon cancer and liver metastasis in colon cancer.

TRPM8 assumes an unanticipated role in colonic motility, presenting a promising target for addressing intestinal functional disorders such as IBS and intestinal spasms [71,72]. In a bid to enhance adenoma detection during colonoscopy, two studies demonstrated the attenuation of colonic peristalsis and abdominal pain following menthol intervention [73,74]. Researchers delved into the mechanism of action, affirming the correlation between hypothermia and colonic peristalsis, a phenomenon absent in TRPM8-deficient mice [30]. In a separate study, researchers initially verified the expression of TRPM8 in the human distal colon. Subsequently, they utilized reverse transcriptase polymerase chain reaction (RT-PCR) and Western blot analysis to validate the presence of TRPM8 gene and protein expression in both the mucosa and smooth muscle layer. Utilizing an array of TRPM8 agonists and inhibitors, ligand-dependent TRPM8 activation was revealed to diminish spontaneous colon movement, with 1-[Diisopropyl-phosphinoyl]-alkane (DIPA)1-8 showing optimal efficacy. TRPM8’s modulation of colonic motility is intertwined with the activation of the large-conductance Ca^2+^-dependent K^+^-channels. Moreover, TRPM8 agonist administration in the stomach induced a heightened colonic temperature, indicating potential involvement in autonomous thermoregulation [31].

The genesis of abdominal pain stemming from the colon is typically attributed to colonic inflammation or sensory afferent nerves. A study documented pain alleviation in IBS patients post-TRPM8 agonist treatment, likely to be attributed to TRPM8’s anti-inflammatory prowess [32]. Andrea M. Harrington and colleagues identified TRPM8 expression in colonic afferent neurons for the first time by means of laser capture microdissection, RT-PCR, immunofluorescence, and retrograde tracing, co-expressed alongside the nociceptive receptor TRPV1, underscoring TRPM8’s potential in assuaging colonic-derived abdominal pain. Intriguingly, TRPM8 was found to exhibit both nociceptive and antinociceptive effects—initially inducing pain before modulating TRPV1 and the TRPA1 nociceptive receptor sensitivity—ultimately curtailing mechanical sensory function and quelling abdominal pain. These two receptors may be independently influenced or there may be cascades associated with TRPV1 and TRPA1 [33]. Similar effects were also observed on the tongue and skin [75,76].

However, other studies have shown that visceral hyperalgesia in colitis may be linked to elevated TRPM8 expression, and the administration of WS-12 can trigger a visceral pain-like response that is counteracted by the TRPM8 inhibitor AMTB hydrochloride. This study posited that the heightened number of TRPM8 nerve fibers in colitis model mice mucosa, coupled with the co-localization of TRPM8 with CGRP and SP in nerve fibers, may underlie this association. This is not completely contradictory to the two-way effect of TRPM8 on pain observed by Andrea M Harrington et al. The disparities in TRPM8’s dual impact on pain warrant further elucidation to effectively harness its anti-inflammatory potential [34]. The pain-modulating influence of menthol suggests a bidirectional pain regulation or activation-threshold-oriented mechanism linked to TRPM8. The findings unveiled a biphasic effect of menthol on sensitivity to cold, with higher concentrations significantly easing pain perception, while lower concentrations yielded no discernible difference [75].

## 5. The Role of TRPM8 in the Liver

Apart from the prostate, the liver exhibits the highest expression of TRPM8 in the human body. TRPM8 not only impacts the liver metastasis of colorectal cancer but also fosters the onset and progression of hepatocellular carcinoma itself [77]. Researchers initially identified the upregulation of TRPM8 expression in hepatocellular carcinoma, correlating with unfavorable pathological attributes. Inhibiting TRPM8 proves effective in curtailing hepatocellular carcinoma development. Subsequent mechanistic inquiries propose that TRPM8 may spur carcinogenesis by inducing nucleolar relative molecule-small nucleolar RNA, H/ACA box 55 (SNORA55) translocation from the nucleus to the mitochondria, thereby modulating ATP synthase F1 α subunit (ATP5A1) and ATP synthase F1 β subunit (ATP5B) to influence mitochondrial function [35]. Another investigation highlighting TRPM8’s advantageous role in liver regeneration posits that TRPM8 boosts hepatocyte renewal through peroxisome proliferator-activated receptor gamma co-activator-1 alpha (PGC1α) modulation, enhancing mitochondrial energy metabolism. This regulatory mechanism may underpin TRPM8’s dual function in both promoting hepatocellular carcinoma cell proliferation and fostering liver regeneration [36].

Studies on liver fibrosis underscore the protective potential of TRPM8 deficiency against inflammation, biliary conditions, and fibrosis, mediated through the S100A9-HNF4α signaling pathway. S100A9 is a member of the S100 protein family, which is released from inflammatory or damaged cells and is strongly associated with various inflammatory and fibrotic diseases, and HNF4α is a major regulator of liver-specific gene expression in fibrogenesis [37]. In summary, TRPM8 consistently exerts a negative impact on liver inflammation, tumors, fibrosis, and beyond, underscoring its potential as a viable target for the future development of TRPM8-targeted therapeutic interventions for liver diseases to mitigate adverse effects.

## 6. The Role of TRPM8 in the Pancreas

In the realm of pancreatic research, cancer has emerged as a prime focus of TRPM8-related investigations. Previous studies have underscored the essential roles of TRPM7 and TRPM8 in normal pancreatic exocrine secretion and carcinogenesis [38]. Notably, anti-TRPM8 immunoreactivity has been discerned in central acinar cells and islet endocrine cells within normal pancreatic tissue. Abnormal TRPM8 expression gradients have been observed in precancerous pancreatic tissues and malignant tumors, with most pancreatic cancers depicting moderate-to-high TRPM8 levels and a positive correlation noted between anti-TRPM8 immunoreactivity and primary tumor size and stage. Silencing TRPM8 through short hairpin RNA in pancreatic cancer cell lines with heightened TRPM8 levels has been shown to hinder invasive capabilities [39]. Clinical analysis has further revealed significantly elevated TRPM8 expression levels in pancreatic cancer tissues compared to non-cancerous controls, with augmented TRPM8 expression closely linked to tumor size, advanced TNM stages, and distant metastasis. Moreover, patients with elevated TRPM8 expression exhibited poorer overall survival (OS) and disease-free survival (DFS) rates [40]. In addition, pancreatic cancer cells lacking TRPM8 showcased diminished proliferation and cell cycle progression capacities alongside heightened cyclin-dependent kinase inhibitor levels, substantiating TRPM8’s indispensable role in cell proliferation within pancreatic adenocarcinoma [41]. Another study also reported abnormal TRPM8 expression in pancreatic cancer tissues that spur cancer by promoting cell cycle progression and impeding cancer cell replicative senescence [9].

The impact of glycosylation on TRPM8 function has been observed in PDA. Studies indicate the presence of unglycosylated TRPM8 protein on the PDA cell membrane, where it functions to inhibit the migration of PDA cells [43]. Another study also noted evidence suggesting that alterations in TRPM8 N-glycosylation in pancreatic cancer cells play a key role in cell proliferation, with unglycosylated TRPM8 potentially offering protective effects in pancreatic cancer, chiefly attributed to its calcium transport capabilities [42]. Models predicting the prognosis of patients with PDA utilizing TRPM8 and related genes have been constructed, underscoring TRPM8’s pivotal pathogenic role in pancreatic tumors and its potential utility as molecular biomarkers and therapeutic targets in pancreatic cancer [44].

In another facet, TRPM8 demonstrates a protective effect against CVB-induced pancreatitis. Recognized as a significant human pathogen with an affinity for pancreatic acinar cells, CVB can evoke mild or severe forms of pancreatitis [78,79]. Observations highlighting the mitigation of CVB infection following hypothermia or menthol treatment culminated in the administration of menthol in CVB-infected mice, resulting in decreased pancreatic viral titers, reduced organ inflammation, and tissue damage mitigation. Notably, TRPM8 silencing reversed these effects, suggesting that the activation of TRPM8 suppresses mitochondrial fission proteins, like dynamin-related protein 1 (DRP1), to counteract TRPV1-induced mitochondrial fission, ultimately leading to a decrease in infection and inflammation [45].

## 7. Conclusions and Outlook

TRPM8 plays a crucial role in a multitude of pathophysiological processes across different digestive system organs, providing substantial research value into the underlying mechanisms. While this article outlined the distinctive role of TRPM8 in various digestive organs, a systematic compilation of its effects across disease classes may be pivotal for harnessing TRPM8 as a clinical drug target. Noteworthy promoting effects have been consistently observed only in cancer studies within the digestive system, spanning esophageal, stomach, colon, liver, and pancreatic cancers. While similar outcomes have been noted in cancer-related studies in other systems, efforts are ongoing to explore relevant therapeutic avenues [3].

Definitive conclusions cannot be drawn for other digestive system diseases due to incomplete research on mechanisms and conflicting findings among studies [22,23,24,25]. In colitis, for instance, although TRPM8 consistently exhibits anti-inflammatory properties across studies, the precise mechanism of its action remains unclear, possibly influenced by study design. While prior research commonly utilized menthol or icilin to stimulate TRPM8, discrepancies in the efficacy of menthol have been noted, potentially linked to the varying concentrations used, leading to discrepancies across studies [75].

Furthermore, classical TRPM8 agonists like menthol or icilin are not exclusively associated with TRPM8. For instance, a study discovered that cholinergic nicotinic receptors serve as the primary molecular targets for menthol-induced gastric relaxation in mice rather than TRP and 5-hydroxytriptamine 3 (5-HT_3_) receptors. The mechanism entails the stimulation of intestinal nerves to decrease acetylcholine release [80]. Additionally, traditional TRPM8 agonists (such as menthol and icilin) may activate other thermosensitive TRP channels (such as TRPV3 and TRPA1) and mutually inhibit TRPM8, thereby limiting the specificity of menthol and icilin as TRPM8 ligands. Consequently, conventional TRPM8 modulators have not been sanctioned as novel cancer treatments due to the emergence of adverse effects observed in initial trials.

Several researchers have examined a series of menthol derivatives (such as CPS-368, CPS-369, CPS-125, WS-5, and WS-12) and determined that these compounds solely target TRPM8 without activating other thermal TRP channels linked to inflammation, including TRPA1, TRPV1, TRPV2, TRPV3, and TRPV4. These TRPM8-specific agonists exhibit heightened potency, efficacy, and specificity compared to menthol. This enhanced specificity offers precise target engagement, which holds substantial promise for future clinical drug development by providing more defined functionalities with fewer side effects [8,81,82]. The utilization of TRPM8-selective agonists, particularly WS-12, has emerged as a primary choice due to its superior selectivity, potency, and effectiveness relative to menthol, without inducing rapid drug resistance observed with icilin-mediated TRPM8 activation [83,84,85]. The strategic adoption and advancement of highly selective TRPM8 agonists and antagonists, alongside the use of genetically modified mice for comprehensive validation, are vital approaches for garnering precise conclusions in the exploration of TRPM8’s pathophysiological role in digestive system disorders while mitigating discrepancies across studies.

However, this does not mean that previous research on menthol has not helped to clarify the function of TRPM8. On the contrary, it may be a shortcut to the truth. The exploration of TRPM8’s analgesic properties was likely sparked by the observed efficacy of menthol as an analgesic [86,87]. While some studies referenced in this review focused solely on the effects of menthol without confirming TRPM8 expression, upon analysis, it becomes apparent that TRPM8 likely serves similar functions. For example, TRPM8 activation can be triggered by agonists like low temperatures or menthol, both of which have been associated with the regulation of esophageal peristalsis during movement, indicating a potential role for TRPM8 in controlling esophageal motility [49,50]. Moreover, in research on menthol’s impact on esophageal inflammation, a consistent inhibitory influence of menthol and TRPM8 on TRPV1 has been noted [56]. By employing highly selective agonists and inhibitors, alongside assessing TRPM8’s potential in ameliorating esophagitis, a deeper understanding of TRPM8’s role in esophageal function can be achieved. It is evident that numerous comparable studies offer valuable insights for further exploration.

By consolidating these insights, TRPM8 emerges as a prospective therapeutic target for managing digestive tract inflammation, tumors, and sensory and functional disorders, showcasing promise as a novel diagnostic, prognostic, and therapeutic marker across esophageal, gastric, and colon cancer research spheres, with substantial potential for future advancement.

## Figures and Tables

**Table 1:** Distribution, functions, and related disorders of TRPM8 channel in the digestive system.

Organs Distributed by TRPM8	Functions	Related Disorders	References
Esophagus	Participating in esophageal sensory transduction	Esophageal sensation and nociception	Yu et al. (2015) [17]
Esophagus	Leading to immune evasion of esophageal cancer cells	Esophageal cancer	Lan et al. (2019) [18]
Stomach	Promoting gastric cancer cell proliferation and metastasis in vivo	Gastric cancer	Xu et al. (2021) [19]
Stomach	As candidates for novel biomarkers in gastric cancer	Gastric cancer	Kong et al. (2023) [20]
Small intestine	Relieving indomethacin-induced small intestinal injury by promoting the expression of calcitonin gene-related peptide (CGRP)	Small intestinal injury	Fouad et al. (2021) [21]
Colon	Inhibiting the role of substance P (SP) in promoting colonic epithelial apoptosis and relieving colitis	Colitis	Zhang et al. (2024) [22]
Colon	Relieving innate inflammatory responses by promoting the expression of CGRP	Colitis	De Jong et al. (2015) [23]
Colon	Suppressing the pro-inflammatory effect of TRPV1 by inhibiting the expression of CGRP	Colitis	Ramachandran et al. (2013) [24]
Colon	Regulating tumor necrosis factor-α (TNF-α) and interleukin (IL)-10 production	Colitis	Khalil et al. (2016) [25]
Colon	Regulating the growth of colorectal cancer cells	Colorectal cancer	Borrelli et al. (2014) [26]
Colon	Regulating tumor development via inhibition of oncogenic Wnt/β-catenin signaling	Colorectal cancer	Pagano et al. (2023) [27]
Colon	Promoting the progression and epithelial–mesenchymal transition (EMT) of colon cancer cells	Colon cancer liver metastasis	Liu et al. (2022) [28]
Colon	Suppressing the EMT of colon cancer cells	Colon cancer liver metastasis	Li et al. (2023) [29]
Colon	Suppressing colonic peristalsis	Irritable bowel syndrome (IBS)	Sugino et al. (2022) [30]
Colon	Reducing colonic spontaneous motility	IBS	Amato et al. (2020) [31]
Colon	Reducing release of inflammatory cytokines IL-1β, IL-6, and TNF-α	IBS	Peiris et al. (2021) [32]
Colon	Desensitizing afferents to mechanical stimulation	Colonic sensation	Harrington et al. (2011) [33]
Colon	Contributing to the visceral hyperalgesia of experimental colitis	Colitis	Hosoya et al. (2014) [34]
Liver	Promoting hepatocellular carcinoma progression	Hepatocellular carcinoma	Fu et al. (2023) [35]
Liver	Contributing to liver regeneration	Hepatectomy	Lei et al. (2022) [36]
Liver	Regulating S100A9 (commonly known as calgranulin B)-hepatocyte nuclear factor 4α (HNF4α) signaling	Inflammation; liver fibrosis	Liu et al. (2022) [37]
Pancreas	As a cancer biomarker and target	Pancreatic adenocarcinoma	Yee et al. (2012) [38]
Pancreas	Related to tumor size/stage and requirement for cancer cell invasion	Pancreatic adenocarcinoma	Yee et al. (2014) [39]
Pancreas	Might be a useful prognostic factor for patients with pancreatic adenocarcinoma	Pancreatic adenocarcinoma	Du et al. (2018) [40]
Pancreas	Being required for cellular proliferation in pancreatic adenocarcinoma	Pancreatic adenocarcinoma	Yee et al. (2010) [41]
Pancreas	Preventing replicative senescence in pancreatic adenocarcinoma	Pancreatic adenocarcinoma	Yee et al. (2012) [9]
Pancreas	Affecting cell proliferation, cell migration, and calcium uptake	Pancreatic adenocarcinoma	Roxana et al. (2017) [42]
Pancreas	Inhibiting pancreatic ductal adenocarcinoma (PDA) cell motility	PDA	Cucu et al. (2014) [43]
Pancreas	Constructing a 5-Gene Model for predicting PDA patient prognosis	PDA	Liu et al. (2023) [44]
Pancreas	Blocking TRPV1-mediated mitochondrial fragmentation following Coxsackie virus B (CVB) exposure and attenuates infection	Pancreatitis	Taylor et al. (2020) [45]
